# Do Mixed-Species Biofilms Dominate in Chronic Infections?–Need for *in situ* Visualization of Bacterial Organization

**DOI:** 10.3389/fcimb.2020.00396

**Published:** 2020-08-05

**Authors:** Lasse Kvich, Mette Burmølle, Thomas Bjarnsholt, Mads Lichtenberg

**Affiliations:** ^1^Department of Immunology and Microbiology, Faculty of Health and Medical Sciences, Costerton Biofilm Center, University of Copenhagen, Copenhagen, Denmark; ^2^Department of Biology, University of Copenhagen, Copenhagen, Denmark; ^3^Department of Clinical Microbiology, Copenhagen University Hospital – Rigshospitalet, Copenhagen, Denmark

**Keywords:** mixed-species biofilm, multi-species biofilm, poly-microbial infections, biofilm, chronic infections

## Abstract

Chronic infections present a serious economic burden to health-care systems. The severity and prevalence of chronic infections are continuously increasing due to an aging population and an elevated number of lifestyle related diseases such as diabetes. Treatment of chronic infections has proven difficult, mainly due to the presence of biofilms that render bacteria more tolerant toward antimicrobials and the host immune response. Chronic infections have been described to harbor several different bacterial species and it has been hypothesized that microscale interactions and mixed-species consortia are present as described for most natural occurring biofilms i.e., aquatic systems and industrial settings, but also for some commensal human biofilms i.e., the mouth microbiota. However, the presence of mixed-species biofilms in chronic infections is most often an assumption based on culture-based methods and/or by means of molecular approaches, such as PCR and sequencing performed from homogenized bulk tissue samples. These methods disregard the spatial organization of the bacterial community and thus valuable information on biofilm aggregate composition, spatial organization, and possible interactions between different species is lost. Hitherto, only few studies have made visual *in situ* presentations of mixed-species biofilms in chronic infections, which is pivotal for the description of bacterial composition, spatial distribution, and interspecies interaction on the microscale. In order for bacteria to interact (synergism, commensalism, mutualism, competition, etc.) they need to be in close proximity to each other on the scale where they can affect e.g., solute concentrations. We argue that visual proof of mixed species biofilms in chronic infections is scarce compared to what is seen in e.g., environmental biofilms and call for a debate on the importance of mixed-species biofilm in chronic infections.

## Introduction

Bacteria in the environment and the human microbiome, including the gut, skin and mouth, are organized in aggregated consortia, also known as biofilms (Bjarnsholt, 2013). Interspecies interactions are well-described among bacteria present in these habitats and have been demonstrated to affect the overall physiology and function of the biofilm, and importantly the host (Burmølle et al., 2014). Biofilms are also present in chronic infections and persistence of the infections is believed to be due to the aggregation of bacteria in protective structures, whereas acute infections are traditionally described to harbor antibiotic susceptible, planktonic bacteria (Burmølle et al., 2010).

In 2010 it was estimated by the National Institutes of Health, that 17 million Americans acquired biofilm infections each year, causing the death of at least 550,000 people in the USA alone (Wolcott and Dowd, 2011). Chronic infections are a substantial burden to patients and to the health-care systems and the economic impact varies depending on the type of chronic infection, e.g., chronic wounds, implant associated infections, cystic fibrosis, etc. An increase in chronic infections is expected in the future due to an aging population, along with an increase in lifestyle diseases such as diabetes, which is one of the major causes of chronic wounds (Narayan et al., 2006; Zimmerli, 2006).

Microbiological diagnosis has traditionally been based on cultivation-dependent methods, which has shown to underestimate the diversity when compared with molecular methods. For instance, a study reported a mixed bacterial flora in 64% of infections using molecular methods and only 10% when examined by cultivation (Xu et al., 2012b). Likewise, other molecular results indicate that poly-microbial infections are much more frequent than previously known where some bacteria, previously never isolated in infections, appear to have important roles (Tatum and Dowd, 2012; Xu et al., 2012b). Molecular studies of chronic wounds indicate that the microbial flora of individual patients is variable, and the spatial distribution of the microorganisms within each wound is heterogeneous (Thomsen et al., 2010; Price et al., 2011; Wolcott et al., 2016). In a study by Thomsen et al. (2010) it was estimated that wounds comprise an average of 5.4 species. However, the role and impact of bacterial co-existence in infections is only now beginning to be identified and though chronic infections might harbor multiple species, it does not *per se* mean that these are found in mixed-species biofilms, nor that they are interacting.

**Figure 1:**
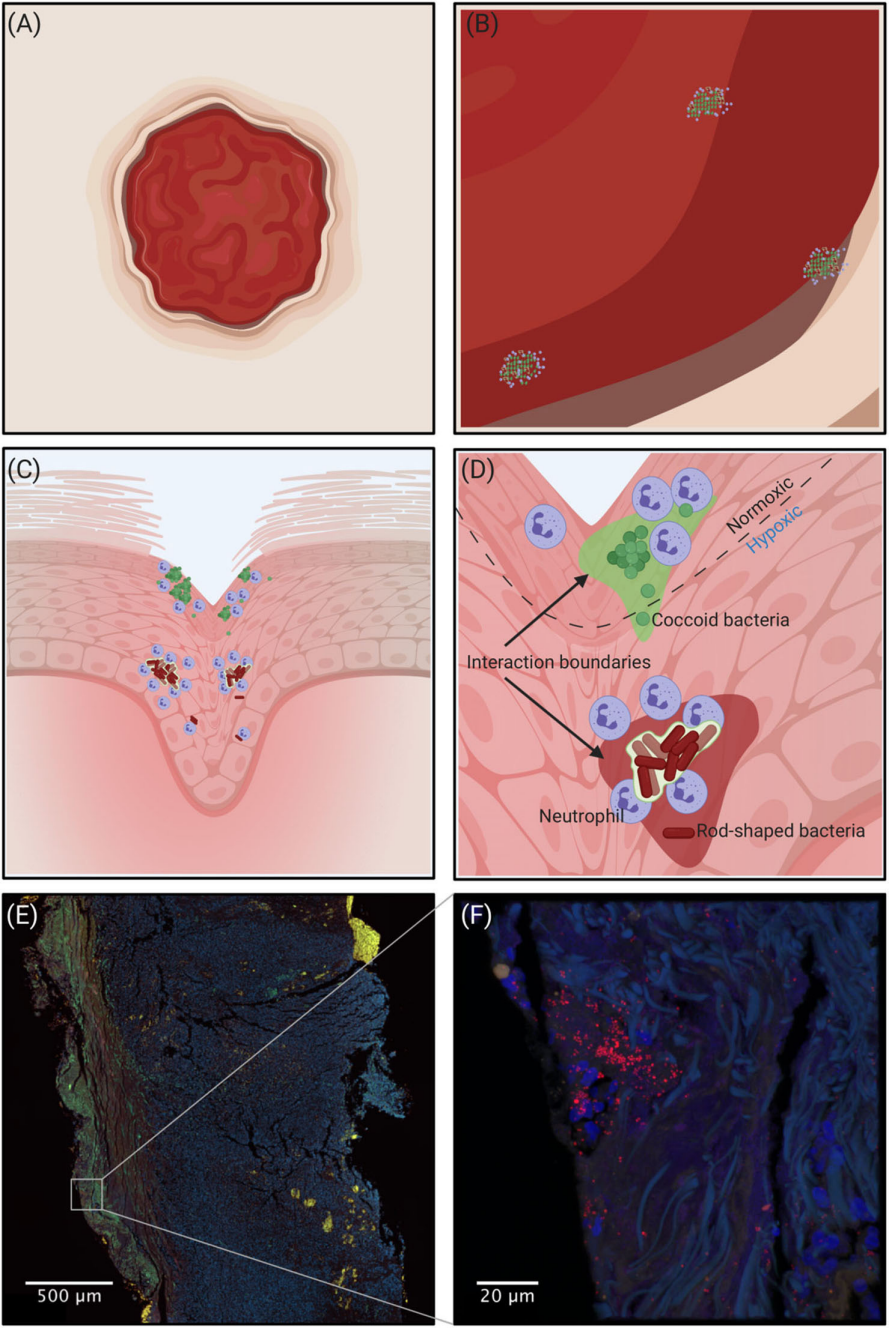
**(A–D)** Conceptual drawing of a wound. Going from macroscopic to microscopic view reveals an increasing heterogeneity in the spatial organization of bacteria depending on the microhabitat. **(A)** Macroscopically it is impossible to identify the regions where bacteria are present. **(B)** Zooming in, bacterial aggregation becomes visible, but without detail of the spatial organization. **(C)** Shows that different species are spatially separated, and **(D)** shows the scale that is more relevant to the bacteria where the microenvironment governs the spatial organization of aerobic and obligate/facultative anaerobic bacteria. The interaction boundaries mark the zone where the individual aggregates can affect the concentration of solutes. **(E,F)** Confocal Laser Scanning Microscopy of a chronic leg ulcer colored by peptide nucleic acid fluorescence *in situ* hybridization (PNA-FISH). A species-specific probe targeting *S. aureus* (red) counter-stained by DAPI to stain the nuclei of human cells (blue) was used to visualize the spatial organization of bacteria in the tissue. Tissue samples were formalin-fixed and paraffin-embedded prior to staining.

**Figure 2:**
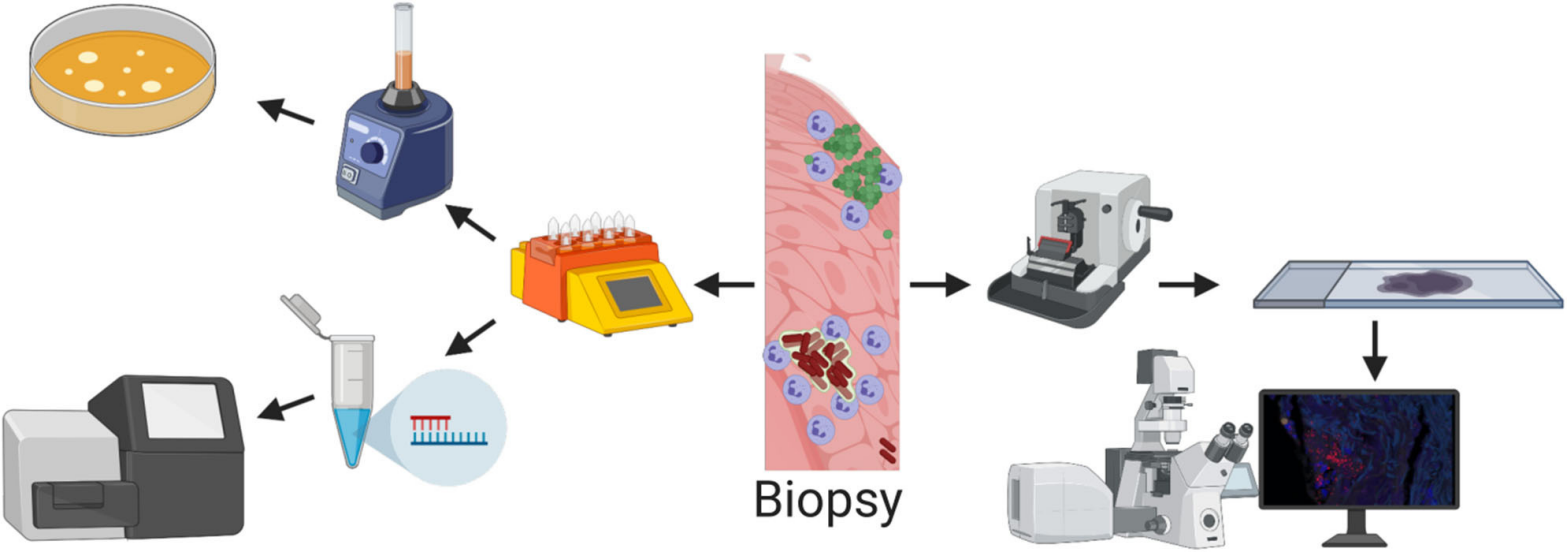
Methods for detecting bacteria from a biopsy. (Top left) Biopsy is homogenized with a tissue dissociator and grown on agar plates. This method is cheap and quick but is very unspecific and the risk of false negative results is high. In addition, spatial structure is lost. (Bottom left) Biopsy is homogenized with a tissue dissociator and DNA is extracted and sequenced. With molecular tools it is possible to find all species present in the biopsy, but all spatial structure is lost. (Right) The spatial structure is retained with imaging techniques such as confocal or electron microscopy where the tissue is sectioned into thin slices. But due to the relatively small area/volume that can be imaged, the risk of not finding the bacteria is high.

To study and verify mixed-species biofilms in chronic infections several innovative molecular techniques can be used. The recent advance in amplicon sequencing using next generation sequencing (NGS) technologies has been successfully used to study bacterial diversity and relative abundance in many biological systems including the human gut microbiome (Qin et al., 2010), necrotizing fasciitis (Rudkjøbing et al., 2016), urinary catheters (Xu et al., 2012a), and prosthetic joint infections (Li M. et al., 2019). Moreover, insight into the *in situ* expression of virulence genes and metabolic pathways at whole genome level has recently become possible via RNA-sequencing (Ibberson and Whiteley, 2019). Additionally, the development of advanced mass spectrometry and high-resolution microscopy (Medini et al., 2008; Eickhardt et al., 2015) has enabled the required quality and amount of data for complete community studies. All methods have advantages and limitations, therefore none of the currently available methods can stand alone when exploring the microbiome of chronic infections. But, when determining whether infections harbor mixed-species biofilms, morphological *in situ* visualization with high-resolution microscopy is essential to verify spatial distribution and co-existence of different species in the same aggregates. A conceptual drawing of a wound and the interaction boundaries between bacteria is visualized in Figure 1 and an example of a *Staphylococcus aureus* infection in a chronic leg ulcer is presented herein, to give an example of a method that leads to the visualization needed to show whether multiple species are present in the same biofilm. In addition, Figure 2 shows some of the methods used to investigate the presence of multiple bacteria in an infection and explains some of their advantages and disadvantages.

In a review by Burmølle et al. (2010) it was highlighted that the newest *in situ* detection and identification techniques indicated low bacterial diversity and overall mono-species biofilms in mixed-species infections. Furthermore, it was noted that those observations were in contrast to what is found in commensal and environmental biofilms (Burmølle et al., 2010). It is pivotal for the treatment of chronic infections to understand the complexity and structure of biofilms and to elucidate whether biofilms consist of intermixed populations. Thus, the first step should be to determine if chronic infections harbor mixed-species biofilms, then we can begin to investigate how different bacteria respond to each other (synergism, commensalism, mutualism, competition, etc.) in the community as well as how they interact with the host and vice versa. Only when we have this information we can begin to treat chronic infections with success, and ratify existing *in vitro* and *in vivo* models.

In this review we define a mixed-species biofilm as an aggregate consisting of different microorganisms interwoven or in close proximity potentially allowing for interactions with each other. These interactions do not need to have any proven advantages. Biofilms that do not meet this definition are here regarded as single-species aggregates. Environmental biofilms might not be comparable to what we can find in humans regarding size, structure, and composition, but literature presenting mixed-species biofilms is included if visual presentation of the above definition is supported. A multilayered biofilm where a stratified community with individual species occupying a certain microenvironment represents an example of an environmental mixed-species biofilm (Schneider et al., 2013; Jørgensen et al., 2019).

This is not a meta-analysis, but rather a comprehensive review of the existing literature. Some studies are excluded i.e., *in vitro* studies showing different species capable of forming mixed-species biofilms, as well as studies using animal models. In addition, we note that not all chronic infections were covered here but rather we focus on some of the most well-described.

We have used PubMed as a search engine using search terms that describe bacterial organization (biofilm, aggregates, fouling, biofouling, or epibiosis) in combination with microscopic methods used to visualize bacteria *in situ* (light microscopy, confocal microscopy, or electron microscopy). These search terms were then used in combination with the topics described in this review. Our aim is to identify and discuss studies that present visual data of mixed-species biofilms in chronic infections and compare this to findings from natural settings and human microbiotas.

## Human Biofilms

Human biofilms are defined as aggregated bacteria that are present in/on our body. These biofilms are further divided into commensal biofilms that co-exist without inducing any harm to our body and biofilms in infections where the intruding microorganisms are unwanted. However, most literature have described the human microbiome during disease (with the exception of the mouth microbiota), so here we present it as biofilm infections seen in human microbiotas as well as specific human chronic infections.

## Microbiotas

### Mouth Microbiota

It is known that dental plaque is a multilayered build-up of microorganisms consisting of many different species that interact with each other and that mechanical removal is necessary to prevent a number of dental diseases such as caries, gingivitis, and periodontitis (Al-Ahmad et al., 2007). In the study by Al-Ahmad the importance of studying dental biofilm-composition *in vivo* was argued, as no model can capture the complexity of the mouth cavity. By using simplistic *in vitro* models, an oversimplification of the conditions in the mouth is easily introduced regarding nutrients, mechanical forces from saliva and presence of unculturable microorganisms (Al-Ahmad et al., 2007). One of the reasons why the oral microbiome is so well-characterized is the ease of introducing and removing implants or foreign bodies which makes *in situ* investigations possible (Kolenbrander et al., 2010; Tytgat et al., 2019). Many studies have used Fluorescent *in situ* Hybridization (FISH) in combination with Confocal Laser Scanning Microscopy (CLSM) to investigate the composition and structure of mixed-species biofilms that are described for dental plaque (Al-Ahmad et al., 2007; Dige et al., 2007; Klug et al., 2011; Karygianni et al., 2012; Dige and Nyvad, 2019). Some studies investigating the structural and bacterial composition in the mouth have used Combinatorial Labeling and Spectral Imaging–Fluorescence *in situ* Hybridization (CLASI-FISH) (Valm et al., 2011) which allows for the investigation of an unprecedented number of different species (Valm et al., 2012). This technique is thus superior to conventional FISH which allows only a few probes to be used at a time. CLASI-FISH seems promising as a method to characterize the structural composition of bacteria in habitats where various microbes reside.

### Vaginal Microbiota

It is well-known that the vaginal microbiota is composed of different acidophilic species. For women in the reproductive age five different communities are described; four of them are mainly composed of *Lactobacillus* spp. while the fifth comprises anaerobes that can be associated with vaginal symptoms, including odor, discharge, and irritation (Smith and Ravel, 2017). Most studies about the vaginal microbiota, where the composition and structure of bacteria has been characterized and visualized, have concerned bacterial vaginosis where the normal flora has shifted toward a more malign flora (Swidsinski et al., 2005; Hardy et al., 2016; Castro et al., 2019).

### Gut Microbiota

The highest concentration of bacteria in our body is in the gut with an estimate of 500–1,000 species. The density varies throughout the gastrointestinal tract where the highest concentration is found in the colon; 10^12^ CFU/g feces (Eckburg et al., 2005; Guarner, 2006). Despite this knowledge, the complexity, diversity, and structure of biofilms is poorly characterized. Stool samples are not suitable to characterize the structure of the biofilms since peristaltic movements rearrange the composition of bacteria throughout the gut. One way to investigate biofilms in the gut would be to study *ex-vivo* tissue samples, though information might get lost due to tissue preparation. Furthermore, sampling from healthy subjects is ethically not permitted due to the invasive techniques used in colonoscopy. These issues bias the current knowledge, which is now predominantly based on tissue samples obtained from people undergoing colonoscopy to get an assessment of an underlying disease.

Normally a homeostatic condition is present in the healthy gut mucosa, where a thick layer of mucus protects the epithelial cells, creating a barrier against the gut microbiota (Li S. et al., 2019). Besides from the vermiform appendix, presence of mixed-species biofilms in the healthy gut mucosa is still debated (Tytgat et al., 2019), though some studies have shown bacterial biofilms in close proximity to the epithelial lining, but only in the appendixes (Palestrant et al., 2004). Presence of biofilms in the colonic crypts has been described for intestinal diseases such as colorectal cancer (Raskov et al., 2018) and visualization of mixed-species biofilms has likewise been reported (Dejea et al., 2014).

### Skin Microbiota

As for the other commensal microbiota described in this review, literature on the healthy skin microbiota is scarce and most studies reporting mixed-species biofilms concern skin diseases. Though sampling is more straightforward some of the same ethical restrictions as described previously, as well as the preservation of structure, hamper the characterization of the healthy skin microbiota when it comes to geographical biofilm composition and structure. Thus, the studies of skin microbiota reported here is mostly derived from cases involving skin diseases. Sapi et al. (2019) investigated the presence of mixed-species biofilms in *Borrelia*-infected human skin biopsies, and found that it was possible to detect *Chlamydia* antigens and DNA in 84% of the sampled *Borrelia*-biofilms. In another study recently published, they found that *Borrelia burgdorferi* and *Helicobacter pylori* was detected in mixed-species biofilms in dermatological skin-samples from people with Morgellons disease (Middelveen et al., 2019). Unfortunately, the morphology of bacteria was not possible to see from the images provided, making it difficult to evaluate the structural composition in those aggregates.

In the studies of Ring et al. (2017) and Bay et al. (2018), CLSM was performed on skin samples obtained from healthy volunteers from moist and dry skin habitats, respectively. The findings revealed that skin microbiota is heterogeneously distributed in bacterial aggregates (aggregate size was scored and ranged from 5 to >50 μm), and that aggregates were most abundant in stratum corneum, the outermost layer of the epidermis, at the infundibulum of hair follicles and within the funnel of hair follicles. A universal- and a coagulase-negative *Staphylococcus* (CoNS) specific PNA-FISH-probe was used in combination of DAPI as a counterstain to stain human nuclei and results did not indicate presence of mixed-species biofilms in either of the skin habitats (Ring et al., 2017; Bay et al., 2018).

To summarize, mixed-species biofilms are highly abundant in the oral cavity, whereas *in situ* evidence for interspecific bacterial mixing in biofilms in/on other human compartments is very scarce.

## Chronic Infections

It has for a long time been recognized that biofilms are responsible for the majority of chronic infections. These include, but are not limited to, otitis, rhinosinusitis, osteomyelitis, chronic pneumonia in cystic fibrosis patients, diabetic foot ulcers, chronic wounds in general, implants, etc. (Burmølle et al., 2010).

### Foreign Materials

Foreign materials inserted into the human body have many times been described to harbor biofilms i.e., catheters, artificial bones and joints, medical devices, etc. Mixed-species biofilm was described for urine catheters some decades ago (Ganderton et al., 1992), although electron microscopy did not show the mixed-species consortia. Instead it was determined according to cultivation, which cannot determine whether the bacteria/biofilms were in close proximity to each other. However, a more recent study has confirmed the presence of mixed-species biofilms on urinary catheters (Stickler, 2008). In a study by Samimi et al. (2013), the presence of biofilm on explanted periorbital material was determined and in 58% of the cases mixed organism growth was reported, but it was not possible to reveal different morphologies by electron microscopy to elucidate whether mixed-species biofilms were present. Jhass et al. (2014) investigated the presence of biofilms on explanted mini-plates and associated screws, where mixed-species biofilms consisting of rods, fusiform, and cocci bacteria were visualized by electron microscopy (Jhass et al., 2014). In a case-study they found that mixed-species biofilms were present on sutures removed from a chronically ill patient that had recurrent infections. They used a mix of specific probes targeting staphylococci species and LIVE/DEAD staining that discriminated between dead and live bacteria. Based on morphological visualization of cocci and rods the authors documented the presence of mixed-species biofilms on this foreign material (Kathju et al., 2009). Mixed-species biofilms have also been reported to grow on prosthetic mesh used for ventral herniorrhaphy (Kathju et al., 2015). Interestingly, implants (screws, stents, pacemaker, etc.) may provide their own niche for bacterial growth and these device-associated bacteria might in future be recognized as an implant associated microbiota (Jakobsen et al., 2018).

### Chronic Wounds

Dermal wounds are often colonized by aerobic and anaerobic bacteria as well as fungi belonging to the normal microbiota of the skin, gut, mouth, or any other microbiota in contact with the wounds (Bertesteanu et al., 2014). Despite the fact that bacteria belonging to several genera are regularly isolated from or detected in wound samples and swaps, only few studies have reported the presence of mixed-species biofilms in diabetic foot ulcers (Johani et al., 2017; Malone et al., 2017; Suryaletha et al., 2018) and chronic pressure ulcers (James et al., 2008). Other studies investigating the presence of bacterial biofilms in diabetic osteomyelitis and chronic venous leg ulcers did not report presence of mixed-species biofilms, though poly-microbial infections were noted (Fazli et al., 2009, 2011; Johani et al., 2019; Malone et al., 2019). Rather, they find a non-random distribution of species occupying different niches in the wound (Fazli et al., 2009).

### Chronic Lung Infections

Patients diagnosed with cystic fibrosis (CF) often suffer from chronic pneumonia. The genetic disorder lead to impaired mucociliary clearance of the viscous mucus allowing inhaled bacteria to colonize the airways. Acute lung infections are often characterized by a number of different bacteria such as *Haemophilus influenza, S. aureus, Burkholderia cepacia* complex, *Stenotrophomonas maltophilia, Streptococcus pneumonia*, and last but not least *Pseudomonas aeruginosa*. If the host immune response fails to clear the acute infection it can develop into a chronic biofilm infection, predominately by *P. aeruginosa* (Rudkjøbing et al., 2012). Although chronic lung infections can be poly-microbial, bacteria are most often found as small mono-species aggregates with no physical interference between different species (Rudkjøbing et al., 2012; Jorth et al., 2015). These findings agree with findings made by Bjarnsholt et al. (2009) where they used PNA FISH probes targeting all bacteria and *P. aeruginosa* specifically in the conductive and respiratory zones of the lungs of chronic *P. aeruginosa* infected CF patients (Bjarnsholt et al., 2009).

### Chronic Otitis Media

Middle ear infections, like chronic suppurative otitis media (CSOM), have for a long time been recognized to harbor biofilms (Homøe et al., 2009). They are characterized by recurrent chronic suppuration with fluctuating silent dry periods. CSOM is further characterized as being a poly-microbial infection consisting of aerobic and anaerobic respiring bacteria (Burmølle et al., 2010). Some studies have reported presence of mixed-species biofilms (Hall-Stoodley et al., 2006; Kania et al., 2008; Hoa et al., 2009; Thornton et al., 2011).

To conclude, mixed-species biofilms have been detected *in situ* in some infections, but for most chronic infections, *in situ* visual evidence of the presence of mixed-biofilms is lacking.

## Environmental Biofilms

Environmental biofilms are defined as aggregated bacteria that are present in natural and industrial settings and are either beneficial or non-favorable seen from an ecological and/or human perspective.

### Biofilms in Food Industry and Industrial Settings

Foodborne diseases have been extensively studied, especially in the last decades where it has become evident that biofilms are present in the food industry (Jahid and Sang-Do, 2014). Biofilms pose a serious challenge in the food industry due to their ability to adhere to many different surfaces and their intrinsic tolerance to disinfectants caused, among others, by the biofilm-matrix protective barrier. Presence of biofilm in the food industry causes food spoilage, equipment damage, increased energy cost and, in worst case, consumer disease (Yuan et al., 2019). In the paper by Yuan et al. (2019) it was highlighted that mixed-species biofilms were the main cause of contamination in the food industry and that studies investigating mixed-species biofilms were needed to understand interspecies interactions and to investigate how these interactions affect biofilm properties that differ from those observed in single-species biofilms (Yuan et al., 2019). Many studies have made use of high-resolution microscopy to confirm the presence of *in situ* mixed-species biofilms on food-surfaces or from surfaces of industrial equipment (Morris et al., 1997; Fett and Cooke, 2003; Rayner et al., 2004; Hassan et al., 2010). With the introduction of high-throughput techniques many have used PCR and sequencing to explore different species in the food industry (Yuan et al., 2019), that might indicate the presence of mixed-species biofilms. Others have used microorganisms isolated from the food environment to make synthetic mixed-species biofilms to investigate the presence and consequences of bacterial interactions (Dominguez-Manzano et al., 2012; Daneshvar Alavi and Truelstrup Hansen, 2013; Gomes et al., 2018).

Biofilms have for a long time been acknowledged in industrial water systems, often referred to as biofouling (Coetser and Cloete, 2005). Biofilms do not necessarily need to be harmful, in some cases they are of crucial importance i.e., in water reclamation and reuse technologies, where biofilms serve as biodegrading systems able to degrade organic contaminants or by decomposing inorganic materials (Bishop, 2007). Though biofilms are beneficial in some settings, they may also be unwanted due to mechanical blockage, degradation (bio-corrosion) of metals, product contamination, and impedance of heat transfer in industrial water systems (Coetser and Cloete, 2005). Biofilms in water systems are often composed of several microorganisms and have been visualized microscopically *in situ* (Møller et al., 1996). Due to difficulties in sampling or *in situ* imaging, several studies have used sludge from reactors or isolated microorganisms from their environment and conducted small-scale synthetic setups reflecting the natural environments to investigate these mixed-species biofilm properties and bacterial interactions (Massol-Deya et al., 1995; Staudt et al., 2004; Fernandez et al., 2008).

### Biofilms in Soil and Aquatic Systems

Biofilms are ubiquitous in the soil and have beneficial impacts on water injection or removal systems, or *in situ* bioremediation (Bishop, 2007). Due to the high number of cells (10^9^ cells g^−1^ soil) and high diversity of bacteria in soil (10^3^–10^6^ species g^−1^ soil) (Torsvik et al., 1990; Gans et al., 2005), the conditions supporting complex consortium of mixed-species biofilms are present. In addition, presence of a diverse microbial life in soil i.e., archaea, actinomycetes, fungi, and algae might also favor inter-kingdom, mixed-species consortia. Nevertheless, little is known concerning the prevalence of mixed-species biofilms in bulk soil, most likely due to difficulties in sampling without disturbing structures (Burmølle et al., 2010; Cai et al., 2019). Sophisticated analysis of soil samples demonstrated that, despite the high abundance of bacteria, the number of neighbors a single bacterium had within an interaction distance of ca. 20 μm was relatively limited (120 cells on average) (Raynaud and Nunan, 2014) and it has been argued that an extreme heterogeneity in the distribution of bacteria is evident at scales more relevant to microorganisms (Vos et al., 2013). Occasional nutrient blooms in the bulk soil, due to addition of organic material for decomposition, e.g., plant material and animal tissue, are likely to result in mixed-species biofilm formation on the relevant material, which would enhance enzyme retention and efficiency, and allow for microbial syntrophy (Jass et al., 2002). Likewise, plant roots are well-known to support the formation of mixed-species biofilms of mutual benefit to the plant host and the microbial community (Pii et al., 2015; Ansari and Ahmad, 2019).

Biofilms in streams such as rivers are commonly composed of not only mixed-species biofilm, but also inter-kingdom mixed biofilms i.e., algae and bacteria. As previously described, *in situ* characterization of the biofilm structure is difficult in environmental samples, so much research is performed by isolating microorganisms from their habitat and using them in laboratory experiments that simulates the habitat from where they were isolated. Results from these experiments have highlighted the complexity of river biofilms, showing microbial diversity in both the base (attached to a substratum) of the biofilm and in the filamentous streamers (floating in the water) that sheds from the biofilms (Besemer et al., 2009). Some studies used advanced microcopy to visualize the presence of mixed-species biofilms. In a study by Trampe et al. (2017), they sampled underwater minerals (ikaite) from a fjord located in Greenland and found that algae (diatoms) and cyanobacteria co-existed on these minerals and were embedded in polymeric substances (Trampe et al., 2017). Biofilms in sediments are also frequently studied. One recent study used a range of FISH techniques to investigate and characterize the microbial- and chemical composition from deep anoxic continental sub-surfaces (Escudero et al., 2018). They observed mixed-species biofilms of bacteria and archaea, indicating that mixed-species biofilms are common and widespread in subsurface environments.

Studies discussed above thus strongly indicate high prevalence of mixed-species biofilms in soil, sub-surfaces, aquatic environments, and industrial settings. However, in many cases the structural integrity is lost during sampling which complicates visual analysis of the spatial organization of microbial biofilms in environmental samples.

## Discussion

### Mixed-Species Infections Increase Virulence in Animal and *in vitro* Studies

Traditional diagnostics and treatment regimens of infections, including chronic wounds, are based on the “one organism—one disease” paradigm. However, chronic infections often harbor many species residing in biofilms, but interactions across species boundaries are poorly understood. The impact of microbial diversity and interspecific interactions on severity and treatment of chronic infections is unclear, but recent experimental studies indicate that the presence of two or more species enhance resistance and virulence compared to single species infections (Holt et al., 2017; Reddinger et al., 2018). In addition, it has been demonstrated in a porcine wound model that acute wounds co-inoculated with *S. aureus* and *P. aeruginosa* healed more slowly than both non-inoculated wounds and those containing only one of the species (Pastar et al., 2013). Likewise, it was shown that Gram-positive strains enhanced the virulence of *P. aeruginosa* in a *Drosophila* model (Korgaonkar et al., 2013). Bacterial-fungal *in vitro* co-culture by *S. aureus* and *Candida albicans* has also been reported to induce enhanced virulence and resistance to host defense mechanisms and antibiotics of *S. aureus* (Harriott and Noverr, 2009; Peters et al., 2010). Although these observations indicate that interspecies interactions in poly-microbial infections are relevant and possible, others argue that the clinical significance of mixed-species biofilms is not clear, and further argue that there is no direct evidence to suggest patients with mixed-species biofilms have less favorable outcomes than those with mono-species biofilms (Johani et al., 2017). Moreover, the role of non-pathogenic species present in infections so far remains unresolved. Recently, there has been an increased focus on fungi in chronic infections including non-healing wounds (Kalan et al., 2016), but, the overall role of fungi in chronic infections is also unresolved and highly debated. In addition, when investigating and elaborating on the impact of multiple species in artificially created chronic infections, in animal models or *in vitro* experiments, these results are only indicative. No model fully reflects the human infectious environment (Pound and Ritskes-Hoitinga, 2018) and the load of bacteria introduced to animal models are in a concentration that probably exceeds what is realistic as compared to chronic infections.

### Visual Evidence of Mixed-Species Biofilms in Chronic Infections Is Scarce

Many studies show that dual- and mixed-species biofilms are possible *in vitro* and that species in mixed biofilms gain advantages compared to those in mono-species biofilms, e.g., elevated protection against disinfectants, protection from grazing (Raghupathi et al., 2017), or stabilization of the microenvironment (Herschend et al., 2018). However, only few have shown that mixed-species biofilms are present *in situ* in for example chronic wounds, mainly due to difficulties in the sampling process and because microscopy cannot be performed *in situ* in many cases. This is true for human as well as environmental biofilms described in this review. Additionally, the “one organism—one disease” mindset in previous investigations has potentially biased against the finding of mixed-species biofilms in chronic infections. Nevertheless, the number of papers that present visual proof of mixed-species biofilms is still surprisingly low, despite an increased access to visualization techniques such as FISH combined with CLSM.

All methods have their limitations, including microscopy. Besides being time-consuming and labor-intensive, fluorescence microscopy only reveals what you are looking for (i.e., when using species specific probes) and is sensitive toward sampling from the right area (i.e., from the area where biofilms are present). As stated in this paper, the only way to visualize the existence of bacteria in a mixed-species biofilm is to use different visualization techniques such as microscopic visualization coupled with molecular approaches, such as FISH and CLSM, or similar (Figure 2). Some of the disadvantages of using FISH for detection of mixed-species biofilms have been reported elsewhere (Costa et al., 2017). Many new molecular methods, combined with microscopy, have evolved over time and are becoming more accessible, resulting in more studies reporting mixed-species biofilms. Omics technologies, combined with microscopy, have evolved over time and are becoming more accessible, resulting in more studies reporting mixed-species biofilms i.e., a study by Liu et al. where they combined FISH with meta-transcriptomic analysis to investigate interspecies interactions reflected in gene expression patterns, relative abundance and spatial organization (Liu et al., 2019). Nevertheless, there is still a need for more studies to elucidate the role of mixed-species biofilms in infections as the existence of mixed-species biofilms, does not *per se* infer any interspecific interaction impacting community properties or function.

### Micro-Environmental Challenges for Mixed-Species Biofilms

Interspecific interactions in mixed biofilms might be neutral, competitive or cooperative (Burmølle et al., 2014). The driving force for bacteria to thrive and interact in mixed-species biofilms must present a beneficial outcome in the form of increased growth or an increased ability of the population to survive. The cases where interactions become competitive will not result in long-term stable mixed-species biofilms, unless the negatively interacting species are physically separated by others. Also, bacteria are present as biofilms when they are in stressful environments to render them protection (Chu et al., 2018; Efimochkina et al., 2018). In many natural settings, or when present in our body, they co-exist with other bacteria and/or archaea and eukaryotic cells. Thus, a biofilm lifestyle (both mono-species and mixed-species) is believed to serve as a strategy to overcome the stressful environment (Jefferson, 2004). Bacteria in the environment, or present in human microbiotas, have evolved over time and may have adapted to a lifestyle where they are dependent on each other to grow and survive. In chronic infections, however, lower numbers and reduced diversity of bacteria are present, which gives fewer opportunities to find suitable collaborators and the time frame possible for co-evolution is most often relatively short. Also, bacteria present in physiological sub-optimal niches, i.e., wounds, encounters multiple stressors, such as the human immune response, while trying to establish a biofilm. We speculate that if not already introduced as a mixed-species biofilm i.e., from the nearby microbiota, a collaboration in this environment seems difficult or happens only sporadically, although a sequential appearance of different species, where only surviving individuals remain at a certain site, could lead to more than one species establishing, growing and interacting at a certain site over time.

Furthermore, the micro-environmental conditions around developing aggregates in infections play a crucial role. Presence or absence of oxygen and the correct nutrients are essential for bacterial growth and biofilm formation (Figure 1). A study demonstrated that *Burkholderia* sp. and *Pseudomonas* sp. form mixed-species biofilms when there was a competition for nutrients, while they developed individual single-species aggregates when nutrients were excessive (Nielsen et al., 2000). Thus, in a wound, where there is access to a warm, moist, and nutritious environment (Bowler et al., 2001) mixed-species biofilms might be rare. In terms of interspecific interactions, the microenvironment also plays a crucial role where the scale at which individuals interact is related to the distance over which they can effect changes in the concentration of gases or solutes (Raynaud and Nunan, 2014). This calling distance have been estimated to ~5–80 μm in a rhizosphere system (Gantner et al., 2006) and by others elegantly shown to occur over very short distances (~1 μm) effectively requiring juxtaposition of different species (Egland et al., 2004). The importance of chemoattractant substances, and thus distance, has also been demonstrated by Limoli et al. (2019), where *S. aureus* and *P. aeruginosa* were used to show that *P. aeruginosa* modifies surface motility according to secreted factors from *S. aureus* (Limoli et al., 2019). Only when *P. aeruginosa* aggregates were at a certain distance of the *S. aureus* aggregates, *P. aeruginosa* responded and changed the surface motility.

### Future Studies

As stated throughout this review, a biofilm can only be categorized as mixed-species if visual presentation shows the presence of different species mixed in the same aggregate and the distances between cells are sufficiently small to allow cell-cell communication. Though some studies have shown presence of mixed-species biofilms in the different chronic infections described in this review, and the number of studies most likely will increase in the future, it seems that mono-species biofilms are dominant in chronic infections. The authors of this review speculate that only when the right species are present at the right time and under conditions favoring mixed-species consortia, mixed-species biofilms can be found. Future studies of mixed-species biofilms should focus not only on visualization but also aim to resolve if *in vivo* mixed-species biofilms actually are synergistic, which could potentially be resolved by e.g., single-cell transcriptomics and metabolomics (Duncan et al., 2019) combined with promising visualization tools, such as CLASI-FISH (Valm et al., 2012).

In addition, with regards to chronic infections, future studies should aim at sampling biopsies where the structural composition of bacteria is maintained by e.g., formalin fixation and paraffin embedment. The use of fixative depends on the type of tissue that is sampled i.e., Carnoy's fixative has been used instead of formalin for the preservation of mucus in colonic tissue, resulting in some elegant structural studies of the bacterial composition in the mucosal layer of colorectal cancer patients (Drewes et al., 2017). Lastly, we note the importance of characterizing the structural composition of the microbiome of healthy individuals as a baseline for the changes seen during infection and disease. Ethical permission to obtain tissue should in future studies, if possible, include tissue from healthy patients undergoing routine investigations where biopsies are already being sampled i.e., during cancer screening or elucidation of underlying diseases.

## Conclusion

Techniques enabling visualization of mixed-species biofilms have been available for several decades now, but still the amount of research presenting visual proof of mixed-species biofilms in chronic infections is scarce. Major challenges when analyzing bacterial spatial organization in human samples are (1) conducting sampling without disturbing the spatial structure, (2) performing *in situ* visualization, and (3) sampling from healthy individuals due to ethical considerations. Also, we only find what we are looking for, which complicates unbiased investigations. To move the field, more detailed investigations of the *in situ* composition and spatial structure of bacteria are warranted.

## Author Contributions

All authors listed have made a substantial, direct and intellectual contribution to the work, and approved it for publication.

## Conflict of Interest

The authors declare that the research was conducted in the absence of any commercial or financial relationships that could be construed as a potential conflict of interest.
